# Supplementary material for: Work, Motherhood, and Nutrition: Investigating the Association of Maternal Employment on Child Nutritional Status in South Asia—A Systematic Review
Source: Nutrients. 2025 Mar 18;17(6):1059. doi: 10.3390/nu17061059 (PMC11946850; doi:10.3390/nu17061059)
Supplement: Supplementary file 1 [file nutrients-17-01059-s001.zip › nutrients-3515898-supplementary.pdf]

Supplementary file

Table S1: Compliance of the observational (Cross-sectional) studies in the present review with STROBE-M checklist

Sr. no.	Scoring scale	Maximum possible Score	Sumon et al. 2023	Win et al. [illegible]	Islam et.al. [illegible]	Huda et al. [illegible]	Sk et al.	Tigga, et al. 2015	Deshmukh, et al. 2013	Yeleswarapu, et al. 2012	Adhikari et al. 2019	Budhathoki et al. 2019	Brauner-Otto, et al.	Khaliq et al 2021	Khan et al. [illegible]	Shinsugi, et al.2019	Galgamuwa, et al 2017
1.1	Study design mentioned. yes=1, no=0	1	1	1	1	1	1	1	1	1	1	1	1	1	1	0	1
1.2	Background explained. yes=1, no=0	1	1	1	1	1	1	1	1	1	1	1	1	1	1	1	1
1.3	Objective mentioned. yes=1, no=0	1	1	0	1	1	1	1	1	1	1	1	1	1	1	1	1
1.4	Participant selection explained. yes=1, no=0	1	1	0	1	0	1	1	1	1	1	1	1	1	1	1	1
1.5	Methods written. yes=1, no=0	1	1	1	1	1	1	1	1	1	1	1	1	1	1	1	1
1.6	Results stated. yes=1, no=0	1	1	1	1	1	1	1	1	1	1	1	1	1	1	1	1
1.7	Conclusions mentioned. yes=1, no=0	1	1	1	1	1	1	1	1	1	1	0	1	1	1	1	1
2.1	Overview of known information. yes=1, no=0	1	1	1	1	1	1	1	1	1	1	1	1	1	1	1	1

**Sr. no.**	Scoring scale	Maximum possible Score	Sumon et al. 2023	Win et al. [illegible]	Islam et.al. [illegible]	Huda et al. [illegible]	Sk et al.	Tigga, et al. 2015	Deshmukh, et al. 2013	Yeleswarapu, et al. 2012	Adhikari et al. 2019	Budhathoki et al. 2019	Brauner-Otto, et al.	Khaliq et al.2021	Khan et al. [illegible]	Shinsugi, et al.2019	Galgamuwa, et al.2017
2.2	Recent pertinent references used. yes=1, no=0	1	1	1	1	1	1	1	1	1	1	1	1	1	1	1	1
2.3	Gaps in current knowledge addressed. yes=1, no=0	1	1	1	1	1	1	1	1	1	1	1	1	1	1	1	1
3.1	Populations specified. yes=1, no=0	1	1	1	1	1	1	1	1	1	1	1	1	1	1	1	1
3.2	Exposures stated. yes=1, no=0	1	1	1	1	1	1	1	1	1	1	1	1	1	1	1	1
3.3	Expected outcomes mentioned. yes=1, no=0	1	1	1	1	1	1	1	1	1	1	1	1	1	1	1	1
3.4	Parameters to be estimated mentioned. yes=1, no=0	1	1	1	1	1	1	1	1	1	1	1	1	1	1	1	1
4.1	Study design explicitely stated.yes=1, no=0	1	1	1	1	1	1	1	1	1	1	1	1	1	1	1	1
4.2	Cohort population	1	1	1	1	1	1	1	1	1	1	1	1	1	1	1	1

**Sr. no.**	Scoring scale	Maximum possible Score	Sumon et al. 2023	Win et al. [illegible]	Islam et.al. [illegible]	Huda et al. [illegible]	Sk et al.	Tigga, et al. 2015	Deshmukh, et al. 2013	Yeleswarapu, et al. 2012	Adhikari et al. 2019	Budhathoki et al. 2019	Brauner-Otto, et al.	Khaliq et al.2021	Khan et al. [illegible]	Shinsugi, et al.2019	Galgamuwa, et al.2017
	explained. yes=1, no=0																
4.3	Institutional Review Board / Ethics committee permission mentioned. Yes=1, no=0	1	1	1	1	0	1	1	1	1	1	1	0	1	1	1	1
4.4	Informed consent taken from participants. Yes=1, no=0	1	0	1	0	1	1	1	1	1	1	1	0	0	1	1	1
5.1	Study setting mentioned. yes=1, no=0	1	1	1	1	1	1	1	1	1	1	1	1	1	1	1	1
5.2	Study location written. yes=1, no=0	1	1	1	1	1	1	1	1	1	1	1	1	1	1	1	1
5.3	Relevant dates mentioned (recruitment, exposure, follow-up, data collection). Yes=1, no=0	4	2	1	2	1	2	0	0	2	2	2	2	2	2	1	1

Sr. no.	Scoring scale	Maximum possible Score	Sumon et al. 2023	Win et al. [illegible]	Islam et.al. [illegible]	Huda et al. [illegible]	Sk et al.	Tigga, et al. 2015	Deshmukh, et al. 2013	Yeleswarapu, et al. 2012	Adhikari et al. 2019	Budhathoki et al. 2019	Brauner-Otto, et al.	Khaliq et al.2021	Khan et al. [illegible]	Shinsugi, et al.2019	Galgamuwa, et al.2017
6,1	Eligibility criteria stated. yes=1, no=0	1	1	1	1	1	1	1	1	1	1	1	1	1	1	1	1
6,2	Sources and methods of selection of participants mentioned. yes=2, only source or method = 1, no=0	2	2	1	2	2	2	2	2	2	2	2	2	2	2	2	2
7.1	Outcome variable/s is/are defined? yes=1, no=0	1	1	1	1	1	1	1	1	1	1	1	1	1	1	1	1
7.2	Exposures defined? yes=1, no=0	1	1	1	1	1	1	1	1	1	1	1	1	1	1	1	1
7.3	Predictors defined? yes=1, no=0	1	1	1	1	0	1	1	0	0	1	1	1	1	1	0	1
7.4	Potential Confounders defined? yes=1, no=0	1	1	1	1	0	1	0	0	0	1	1	0	1	1	0	0

**Sr. no.**	Scoring scale	Maximum possible Score	Sumon et al. 2023	Win et al.	Islam et.al.	Huda et al.	Sk et al.	Tigga, et al.	Deshmukh, et al. 2013	Yeleswarapu, et al. 2012	Adhikari et al. 2019	Budhathoki et al. 2019	Brauner-Otto, et al.	Khaliq et	Khan et al.	Shinsugi, et al.2019	Galgamuwa, et
7.5	Effect Modifiers defined? yes=1, no=0	1	1	0	0	0	0	0	0	0	0	0	0	1	0	0	0
7.6	Diagnostic Criteria defined? yes=1, no=0	1	1	1	1	1	1	1	1	1	1	1	1	1	1	1	1
8.1	For each variable of interest sources of data given. yes=1, no=0	1	1	1	1	1	1	1	1	1	1	1	1	1	1	1	1
8.2	For each variable of interest methods of assessment (measurement) mentioned. yes=1, no=0	1	1	1	1	1	1	1	1	1	1	1	1	1		1	1
8.3	For each variable of interest comparability of assessment methods described (If there is more than one group). yes=1, no=0	1	1	1	1	1	1	1	1	1	1	1	1	1	1	1	1

**Sr. no.**	Scoring scale	Maximum possible Score	Sumon et al. 2023	Win et al.	Islam et.al.	Huda et al.	Sk et al.	Tigga, et al.	Deshmukh, et al. 2013	Yeleswarapu, et al. 2012	Adhikari et al. 2019	Budhathoki et al. 2019	Brauner-Otto, et al.	Khaliq et	Khan et al.	Shinsugi, et al.2019	Galgamuwa, et
9	Efforts to address potential sources of bias described. yes=1, no=0	1	0	0	0	0	0	0	0	0	0	0	1	0	0	0	0
10.1	Known prevalence of variable from literature stated. yes=1, no=0	1	0	0	0	0	1	0	0	0	1	1	1	0	0	0	0
10.2	Required significance mentioned. yes=1, no=0	1	0	1	0	1	0	1	0	0	0	0	0	0	0	1	1
10.3	Required power mentioned. yes=1, no=0	NA	0	NA	1	NA	0	NA	NA	NA	0	0	0	0	0	NA	NA
11.1	Explained how quantitative variables were handled in the analyses. yes=1, no=0	1	1	1	1	1	1	1	1	1	1	1	1	1	1	1	1
11.2	Described which groupings were chosen. yes=1, no=0	1	1	1	1	1	1	1	1	1	1	1	1	1	1	1	1

**Sr. no.**	Scoring scale	Maximum possible Score	Sumon et al. 2023	Win et al. [illegible]	Islam et.al. [illegible]	Huda et al. [illegible]	Sk et al.	Tigga, et al. 2015	Deshmukh, et al. 2013	Yeleswarapu, et al. 2012	Adhikari et al. 2019	Budhathoki et al. 2019	Brauner-Otto, et al.	Khaliq et al.2021	Khan et al. [illegible]	Shinsugi, et al.2019	Galgamuwa, et al.2017
11. 3	Described why specific grouping were chosen. yes=1, no=0	1	1	1	1	1	1	1	1	1	1	1	1	1	1	1	1
12. 1	Statistical methods, including those used to control for confounding explained. yes=1, no=0	1	1	1	1	0	1	1	1	0	1	1	1	1	1	1	1
12. 2	Methods used to examine subgroups and interactions explained. yes=1, no=0	1	1	1	1	0	1	0	1	0	1	1	1	1	1	1	1
12. 3	Method to handle missing data addressed. yes=1, no=0	1	0	1	0	0	1	0	0	0	0	0	1	0	0	1	0
12. 4	For cross-sectional study, described analytical methods taking account of	1	1	1	1	1	1	1	1	1	1	1	1	1	1	1	1

**Sr. no.**	Scoring scale	Maximum possible Score	Sumon et al. 2023	Win et al. [illegible]	Islam et.al. [illegible]	Huda et al. [illegible]	Sk et al.	Tigga, et al. 2015	Deshmukh, et al. 2013	Yeleswarapu, et al. 2012	Adhikari et al. 2019	Budhathoki et al. 2019	Brauner-Otto, et al.	Khaliq et al.2021	Khan et al. [illegible]	Shinsugi, et al.2019	Galgamuwa, et al.2017
	sampling strategy. yes=1, no=0																
12.5	Sensitivity analyses described. yes=1, no=0	1	0	0	0	0	0	0	0	0	0	0	0	1	0	0	0
13.1	Numbers of eligible Individuals reported. yes=1, no=0	1	1	1	1	1	1	1	1	1	1	1	1	1	1	1	1
13.2	Number of individuals Included in the Study reported. yes=1, no=0	1	1	1	1	1	1	1	1	1	1	1	1	1	1	1	1
13.4	Number of individuals analyzed reported. yes=1, no=0	1	1	1	1	1	0	1	1	1	1	1	1	1	1	1	1
13.5	Give Reasons for Non-participation at each stage. yes=1, no=0	1	1	0	1	0	0	1	1	0	0	0	0	1	0	1	0

**Sr. no.**	Scoring scale	Maximum possible Score	Sumon et al. 2023	Win et al. [illegible]	Islam et.al. [illegible]	Huda et al. [illegible]	Sk et al.	Tigga, et al. 2015	Deshmukh, et al. 2013	Yeleswarapu, et al. 2012	Adhikari et al. 2019	Budhathoki et al. 2019	Brauner-Otto, et al.	Khaliq et al.2021	Khan et al. [illegible]	Shinsugi, et al.2019	Galgamuwa, et al.2017
13.6	Recruitment and follow-up flow diagram presented. yes=1, no=0	1	0	0	1	0	0	0	0	0	0	0	0	1	0	1	0
14.1	Characteristics (eg, Demographic, Clinical, Social) of study participants written. yes=1, no=0	1	1	1	1	1	1	0	0	0	1	1	0	1	1	1	1
14.2	Information on potential confounders written. yes=1, no=0	1	1	1	1	0	1	0	0	0	1	0	0	1	0	0	0
14.3	Number of participants with missing data for each variable of interest indicated. yes=1, no=0	1	0	1	0	1	1	0	0	0	1	1	1	1	0	1	0
15	Cross-sectional Study: Numbers of outcome	1	1	1	1	1	1	1	1	1	1	1	1	1	1	1	1

**Sr. no.**	Scoring scale	Maximum possible Score	Sumon et al. 2023	Win et al. [illegible]	Islam et.al. [illegible]	Huda et al. [illegible]	Sk et al.	Tigga, et al. 2015	Deshmukh, et al. 2013	Yeleswarapu, et al. 2012	Adhikari et al. 2019	Budhathoki et al. 2019	Brauner-Otto, et al.	Khaliq et al.2021	Khan et al. [illegible]	Shinsugi, et al.2019	Galgamuwa, et al.2017
	events or summary measures reported. yes=1, no=0																
16.1	Given unadjusted estimates with precision (confidence interval). yes=1, no=0	1	1	1	1	1	1	1	1	1	1	1	1	1	1	1	1
16.2	Given confounder-adjusted estimates (confidence interval). yes=1, no=0	1	1	1	1	0	1	0	0	0	1	1	0	1	1	1	0
16.3	Described which confounders were adjusted for and why they were Included. yes=1, no=0	1	0	1	1	0	1	0	0	0	1	1	0	1	1	0	0
16.4	Reported category	1	1	1	1	1	1	1	1	1	1	1	1	1	1	1	1

**Sr. no.**	Scoring scale	Maximum possible Score	Sumon et al. 2023	Win et al.	Islam et.al.	Huda et al.	Sk et al.	Tigga, et al.	Deshmukh, et al. 2013	Yeleswarapu, et al. 2012	Adhikari et al. 2019	Budhathoki et al. 2019	Brauner-Otto, et al.	Khaliq et al.2021	Khan et al.	Shinsugi, et al.2019	Galgamuwa, et al.2017
	boundaries when continuous variables were categorized. yes=1, no=0																
16.5	Reported estimates of relative risk into absolute risk for a meaningful time period. yes=1, no=0	1	0	0	0	0	0	0	0	0	0	0	0	0	0	0	0
17	Reported other analyses done—e.g., analyses of subgroups and interactions, and sensitivity analyses. yes=1, no=0	1	0	1	0	0	0	0	0	0	0	0	0	1	0	1	0
18	Summarized key results with reference to study objectives. yes=1, no=0	1	0	1	1	1	1	1	1	1	1	1	1	0	1	1	1

**Sr. no.**	Scoring scale	Maximum possible Score	Sumon et al. 2023	Win et al.	Islam et.al.	Huda et al.	Sk et al.	Tigga, et al.	Deshmukh, et al. 2013	Yeleswarapu, et al. 2012	Adhikari et al. 2019	Budhathoki et al. 2019	Brauner-Otto, et al.	Khaliq et al.	Khan et al.	Shinsugi, et al.2019	Galgamuwa, et
19.1	Discussed study limitations. yes=1, no=0	1	1	1	1	1	1	0	0	0	1	1	1	1	1	1	1
19.2	Discussed direction and magnitude of potential bias. yes=1, no=0	2	1	0	1	0	0	0	0	0	0	0	1	1	0	0	0
20.1	Presented a cautious overall interpretation considering objectives, yes=1, no=0	1	1	1	1	1	1	1	1	1	1	1	1	1	1	1	1
20.2	Presented a cautious overall interpretation considering limitations, yes=1, no=0	1	1	1	1	1	1	0	0	0	1	1	1	1	1	1	1
20.3	Presented a cautious overall interpretation considering multiplicity of analyses, yes=1, no=0	1	1	1	1	1	1	1	1	1	1	1	1	1	1	1	1

**Sr. no.**	Scoring scale	Maximum possible Score	Sumon et al. 2023	Win et al.	Islam et.al.	Huda et al.	Sk et al.	Tigga, et al.	Deshmukh, et al. 2013	Yeleswarapu, et al. 2012	Adhikari et al. 2019	Budhathoki et al. 2019	Brauner-Otto, et al.	Khaliq et al.2021	Khan et al.	Shinsugi, et al.2019	Galgamuwa, et al.2017
20.4	Presented a cautious overall interpretation considering results from Similar Studies, yes=1, no=0	1	1	1	1	1	1	1	1	1	1	1	1	1	1	1	1
20.5	Presented a cautious overall interpretation considering and other Relevant Evidence yes=1, no=0	1	1	1	1	1	1	1	1	1	1	1	1	1	1	1	1
21	Discussed the generalizability (external validity) of the study results. yes=1, no=0	1	1	1	1	0	0	0	0	0	0	0	0	1	0	0	0
22.1	Given the source of funding. yes=1, no=0	1	1	1	0	1	1	1	0	0	1	1	0	1	1	1	1
22.2	Given the role of the funders for the present	1	1	0	0	0	1	0	0	0	1	1	0	1	1	1	0

Sr. no.	Scoring scale	Maximum possible Score	Sumon et al. 2023	Win et al.	Islam et.al.	Huda et al.	Sk et al.	Tigga, et al.	Deshmukh, et al. 2013	Yeleswarapu, et al. 2012	Adhikari et al. 2019	Budhathoki et al. 2019	Brauner-Otto, et al.	Khaliq et al 2021	Khan et al.	Shinsugi, et al.2019	Galgamuwa, et al 2017
	study. yes=1, no=0																
22.3	Stated author's and co-authors contributions. yes=1, no=0	1	1	1	1	1	1	0	0	0	1	1	0	1	1	1	1
22.4	Given a statement on data available online. yes=1, no=0	1	1	1	1	0	1	0	0	0	1	1	0	1	1	1	1
22.5	Given a statement on competing interests. yes=1, no=0	1	1	1	1	1	1	0	1	1	1	1	1	1	1	1	1
	Total	**77**	62	61	63	51	63	49	48	47	63	61	55	67	59	61	56
	percentage compliance	100	80.51	79.22	81.81	66.23	81.81	63.63	62.33	61.03	81.81	79.22	71.42	87.01	76.62	79.22	72.72

Table S2: Compliance of the observational (Case-control) studies in the present review with STROBE-M checklist

Sr.no.	Scoring scale	Maximum possible Score	Sigdel et al. 2020	Hossain et al. 2020	Ambadekar et al. 2017	Iftikhar et al. 2017
1.1	Study design mentioned. yes=1, no=0	1	1	1	1	1
1.2	Background explained. yes=1, no=0	1	1	1	1	0
1.3	Objective mentioned. yes=1, no=0	1	1	1	1	1
1.4	Participant selection explained. yes=1, no=0	1	1	1	1	1
1.5	Methods written. yes=1, no=0	1	1	1	1	1
1.6	Results stated. yes=1, no=0	1	1	1	1	1
1.7	Conclusions mentioned. yes=1, no=0	1	1	1	1	1
2.1	Overview of known information. yes=1, no=0	1	1	1	1	1
2.2	Recent pertinent references used. yes=1, no=0	1	1	1	1	1
2.3	Gaps in current knowledge addressed. yes=1, no=0	1	1	1	1	1
3.1	Populations specified. yes=1, no=0	1	1	1	1	1
3.2	Exposures stated. yes=1, no=0	1	1	1	1	1
3.3	Expected outcomes mentioned. yes=1, no=0	1	1	1	1	1
3.4	Parameters to be estimated mentioned. yes=1, no=0	1	1	1	1	1
4.1	Study design explicitly stated. Yes=1, no=0	1	1	1	1	1
4.2	Cohort population explained. yes=1, no=0	1	1	1	1	1
4.3	Follow up time period stated. yes=1, no=0	1	0	1	1	1

Sr.no.	Scoring scale	Maximum possible Score	Sigdel et al. 2020	Hossain et al. 2020	Ambadekar et al. 2017	Iftikhar et al. 2017
4.4	Institutional Review Board / Ethics committee permission mentioned. Yes=1, no=0	1	1	1	1	1
4.5	Informed consent taken from participants. Yes=1, no=0	1	1	1	0	1
5.1	Study setting mentioned. yes=1, no=0	1	1	1	1	1
5.2	Study location written. yes=1, no=0	1	1	1	1	1
5.3	Relevant dates mentioned (recruitment, exposure, follow-up, data collection). All 4 dates mentioned=4, few dates mentioned (1 to 3) = 1 to 3, no=0	2	0	2	2	1
6,1 Case control	Eligibility criteria stated. yes=1, no=0	1	1	1	1	1
6,2 Case Contro l	Sources and methods of case ascertainment and control selection written. yes=2, only source or method = 1, no=0	2	2	2	2	1
6,3 Case control	Rationale for the choice of cases and controls stated. yes=1, no=0	1	1	1	1	1
6,4 Case control	For matched studies give matching criteria. yes=1, no=0	1	1	0	1	1
6,5 Case control	For matched studies give number of controls per case. yes=1, no=0	1	1	0	1	1
7.1	Outcome variable/s is/are defined? yes=1, no=0	1	1	1	1	1
7.2	Exposures defined? yes=1, no=0	1	1	1	1	1
7.3	Predictors defined? yes=1, no=0	1	0	1	0	1

Sr.no.	Scoring scale	Maximum possible Score	Sigdel et al. 2020	Hossain et al. 2020	Ambadekar et al. 2017	Iftikhar et al. 2017
7.4	Potential Confounders defined? yes=1, no=0	1	0	0	0	1
7.5	Effect Modifiers defined? yes=1, no=0	1	0	0	0	0
7.6	Diagnostic Criteria defined? yes=1, no=0	1	1	1	1	1
8.1	For each variable of interest sources of data given. yes=1, no=0	1	1	1	1	1
8.2	For each variable of interest methods of Assessment (measurement) mentioned. yes=1, no=0	1	1	1	1	1
8.3	For each variable of interest Comparability of assessment methods described (If there is more than one group). yes=1, no=0	1	1	1	1	1
9	Efforts to address potential sources of bias described. yes=1, no=0	1	0	0	1	0
10.1	Known prevalence of variable from literature stated. yes=1, no=0	1	1	0	0	0
10.2	Required significance mentioned. yes=1, no=0	1	1	1	1	1
10.3	Required power mentioned. yes=1, no=0	1	1	1	1	0
11.1	Explained how quantitative variables were handled in the analyses. yes=1, no=0	1	1	1	1	1
11.2	Described which groupings were chosen. yes=1, no=0	1	1	1	1	1
11.3	Described Why specific grouping were chosen. yes=1, no=0	1	1	1	1	1
12.1	Statistical methods, including those used to control for confounding explained. yes=1, no=0	1	0	1	0	1
12.2	Methods used to examine subgroups and interactions explained. yes=1, no=0	1	1	1	1	1

Sr.no.	Scoring scale	Maximum possible Score	Sigdel et al. 2020	Hossain et al. 2020	Ambadekar et al. 2017	Iftikhar et al. 2017
12.3	Method to handle missing data addressed. yes=1, no=0	1	0	0	0	0
12.4	For Case-control study, explained how matching of cases and controls was addressed. yes=1, no=0	1	1	0	1	1
12.5	Sensitivity analyses described. yes=1, no=0	1	0	0	0	0
13.1	13.1 Numbers of eligible Individuals reported. yes=1, no=0	1	1	1	1	1
13.2	13.2 Number of individuals Included in the Study reported. yes=1, no=0,	1	1	1	1	1
13.3	Number of individuals completing follow-up reported. yes=1, no=0	1	1	1	1	1
13.4	Number of individuals analyzed reported. yes=1, no=0	1	1	1	1	1
13.5	Give Reasons for Non-participation at each stage. yes=1, no=0	1	1	0	1	0
13.6	Recruitment and follow-up flow diagram presented. yes=1, no=0	1	1	0	0	0
14.1	Characteristics (eg, Demographic, Clinical, Social) of study participants written. yes=1, no=0	1	1	0	0	1
14.2	Information on exposures and potential confounders written. yes=1, no=0	1	1	1	1	1
14.3	Number of participants with missing data for each variable of interest indicated. yes=1, no=0	1	0	0	0	0
15	Case-control Study: Numbers in each exposure category, or summary measures of exposure reported. yes=1, no=0	1	1	1	0	1

Sr.no.	Scoring scale	Maximum possible Score	Sigdel et al. 2020	Hossain et al. 2020	Ambadekar et al. 2017	Iftikhar et al. 2017
16.1	Given unadjusted estimates with precision (confidence interval). yes=1, no=0, NA	1	1	1	1	1
16.2	Given confounder-adjusted estimates (confidence interval). yes=1, no=0	1	1	1	0	1
16.3	Described which confounders were adjusted for and why they were Included. yes=1, no=0	1	0	0	0	1
16.4	Reported category boundaries when continuous variables were categorized.	1	1	1	1	1
16.5	Reported estimates of relative risk into absolute risk for a meaningful time period. yes=1, no=0	1	0	0	0	0
17	Reported other analyses done—eg, analyses of subgroups and interactions, and sensitivity analyses. yes=1, no=0	1	0	0	1	0
18	Summarized key results with reference to study objectives. yes=1, no=0	1	1	1	1	1
19.1	Discussed study limitations. yes=1, no=0	1	1	1	0	0
19.2	Discussed direction and magnitude of potential bias. yes=1, no=0	2	0	1	1	0
20.1	Presented a cautious overall interpretation considering objectives, yes=1, no=0	1	1	1	1	1
20.2	Presented a cautious overall interpretation considering limitations, yes=1, no=0	1	1	1	0	0
20.3	Presented a cautious overall interpretation considering multiplicity of Analyses, yes=1, no=0	1	0	1	0	0
20.4	Presented a cautious overall interpretation considering results from Similar Studies, yes=1, no=0	1	1	1	1	1

Sr.no.	Scoring scale	Maximum possible Score	Sigdel et al. 2020	Hossain et al. 2020	Ambadekar et al. 2017	Iftikhar et al. 2017
20.5	Presented a cautious overall interpretation considering and other Relevant Evidence yes=1, no=0	1	1	1	1	1
21	Discussed the generalizability (external validity) of the study results. yes=1, no=0	1	1	0	0	0
22.1	Given the source of funding. yes=1, no=0	1	1	1	1	1
22.2	Given the role of the funders for the present study. yes=1, no=0	1	0	0	0	0
22.3	Stated author's and co-authors contributions. yes=1, no=0	1	1	1	0	1
22.4	Given a statement on data available online. yes=1, no=0	1	1	0	0	0
22.5	Given a statement on competing interests. yes=1, no=0	1	1	1	1	1
	Total	**81**	**63**	**61**	**58**	**59**
	percentage compliance	100	77.77	75.3	71.6	72.83
